# Spontaneous Postpartum Cecal Perforation After Vaginal Delivery: An Unexpected Life-Threatening Complication of Ogilvie's Syndrome

**DOI:** 10.7759/cureus.111182

**Published:** 2026-06-19

**Authors:** Audipudi Swetha, Ajith Kumar A K, Namita Sinha Verma, Mohammed Rameez K, Nikhil N

**Affiliations:** 1 Critical Care Medicine, Aster Hospital Whitefield, Bengaluru, IND; 2 Radiology, Aster Hospital Whitefield, Bengaluru, IND

**Keywords:** caecal perforation, carbapenem-resistant enterobacteriaceae (cre), ogilvie's syndrome, paralytic ileus, systemic inflammatory response syndrome (sirs)

## Abstract

Acute colonic pseudo-obstruction (Ogilvie syndrome) is one of the rare but potentially fatal post-partum complications characterized by massive colonic dilation without mechanical obstruction. Cecal perforation secondary to Ogilvie syndrome is rarer and is associated with high morbidity and mortality. While more common after cesarean sections, its occurrence following vaginal delivery is extremely rare, and there are only a couple of case reports in the medical literature. Lack of awareness of these life-threatening post-partum complications among treating physicians can lead to unacceptable diagnostic delays, high morbidity, and mortality. Here, we report one such case of post-partum Ogilvie syndrome after vaginal delivery, which progressed to cecal perforation requiring explorative laparotomy, right hemicolectomy, and ileostomy. We intend to highlight the importance of these life-threatening complications, for which enhanced awareness, a high degree of suspicion with anticipation, and early diagnosis can help prevent progression to further complications. Prompt surgical intervention offers a better clinical outcome in these patients with cecal perforation.

## Introduction

Ogilvie syndrome predominantly affects hospitalized or postoperative patients and is frequently associated with severe medical illness, electrolyte disturbances, trauma, infections, neurological disorders, and recent surgical interventions [1,2]. About 10% of all reported cases are related to Obstetric and Gynaecological procedures, with cesarean section being the most common associated factor [3,4]. In the postpartum setting, Ogilvie syndrome is reported more frequently following cesarean section [5], likely due to increased surgical manipulation, immobility, autonomic dysfunction, and use of regional anesthesia. The syndrome may easily be overlooked because abdominal discomfort, bloating, and altered bowel habits are often attributed to normal postpartum recovery [6]. This diagnostic challenge may delay timely intervention and predispose patients to catastrophic complications, including bowel ischemia, necrosis, and cecal perforation.

Among the complications of Ogilvie syndrome, large bowel perforation represents the most feared and life-threatening event, carrying significant maternal morbidity and mortality due to fecal peritonitis and sepsis [7]. Early recognition of persistent abdominal distension and systemic inflammatory signs is therefore critical in preventing progression to perforation [8]. A high index of clinical suspicion and a thorough clinical assessment of the gastrointestinal system in the post-surgical patient are essential for the management of this serious complication [9]. Only a limited number of cases describing postpartum cecal perforation secondary to Ogilvie syndrome following vaginal delivery have been reported in the literature to date [10,11]. We present a rare case of postpartum Ogilvie syndrome after forceps-assisted vaginal delivery in a previously healthy primigravida, which rapidly progressed to spontaneous cecal perforation requiring emergency laparotomy, bowel resection, and diversion ileostomy. This report aims to emphasize the importance of maintaining a high index of suspicion for Ogilvie syndrome even after non-cesarean deliveries, and to highlight the need for early diagnosis, close inpatient monitoring, serial imaging, and timely surgical intervention so as to reduce the maternal morbidity and mortality.

## Case presentation

History

A 31-year-old healthy primigravida with cephalic presentation underwent forceps-assisted vaginal delivery (with right mediolateral episiotomy) under epidural analgesia on her expected date of delivery at 40 weeks of gestation. She was asymptomatic at presentation (she came for induction of labor) except for a well appreciation of fetal movements. She delivered a healthy baby weighing 4.3 Kg with a good APGAR score. About 12 hours after delivery, she developed abdominal pain, nausea, and vomiting with significant bloating of the abdomen. General surgery consultation was obtained, and a provisional diagnosis of “ileus” was made. She was medically managed with nil per oral, IV fluids, and was mobilized. There was no history of pre-existing constipation or evidence of any significant dys-electrolytrolemia.

Her abdominal symptoms were managed medically with proton pump inhibitors, as needed, and anti-emetics (metoclopramide plus ondansetron). On postpartum day 2, the pain subsided, and bowels were opened. Distension still persisted. On postpartum day 3, she had fever spikes, tachycardia, and persistent abdominal distension, though she remained hemodynamically stable, with no respiratory distress and oxygenation-wise stable. The episiotomy site was clean. Breast local examination did not reveal any features of mastitis. Intravenous (IV) amoxicillin-clavulanic acid was administered by the obstetrician, and the patient was subjected to imaging. Imaging is shown below, X-ray abdomen erect (Figure 1), and a Contrast Enhanced Computed Tomography (CECT) (Figures 2, 3) confirmed features of pseudo-colonic obstruction, dilated large bowel loops with no obvious transition point, contracted sigmoid colon) (Figures 2, 3). The cecal diameter was 7.2 cm.

**Figure 1:**
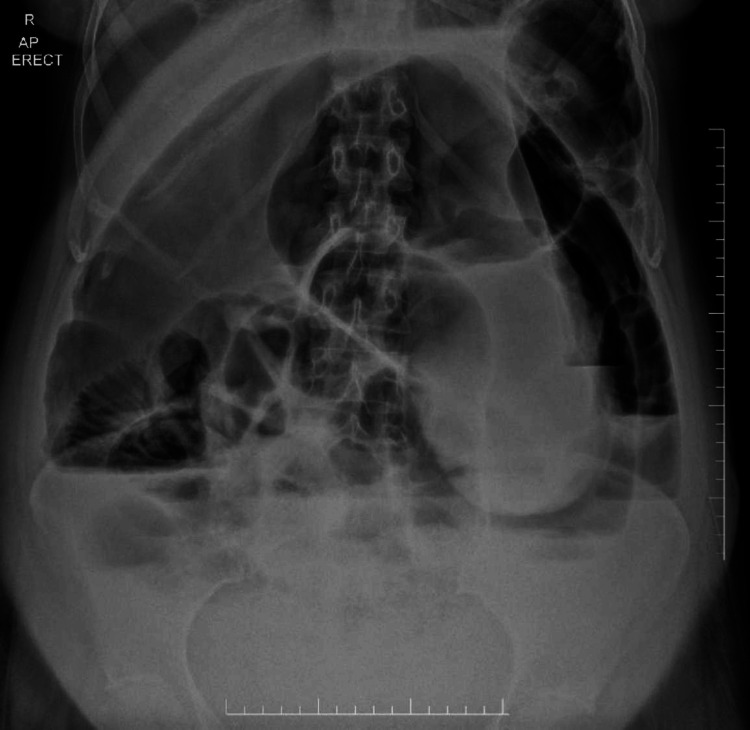
X-ray abdomen (erect)

**Figure 2:**
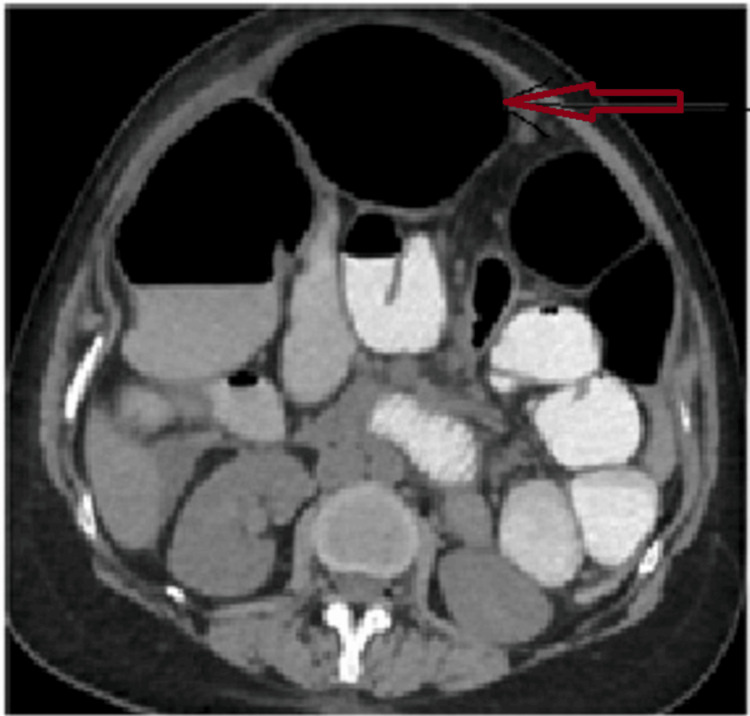
CT abdomen with oral contrast on postpartum day 3 Dilated small and large bowel loops with delayed contrast passage were observed.

**Figure 3:**
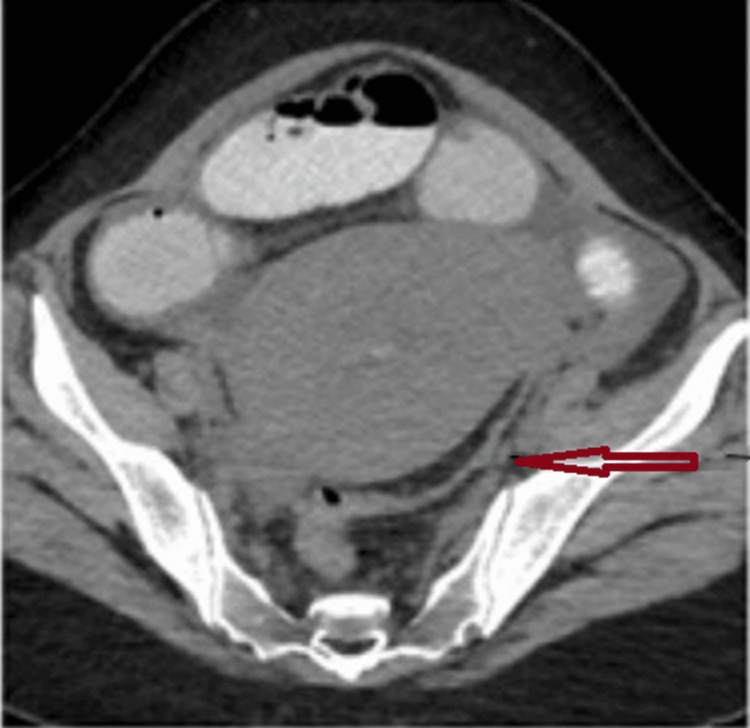
CT abdomen with oral contrast on postpartum day 3 The red arrow is showing a contracted sigmoid colon.

Though the ongoing medical management appeared successful with the return of bowel function &reduction of pain, the fever spikes persisted. She remained hemodynamically stable. She was advised to undergo further monitoring and evaluation in the hospital. However, the patient refused further monitoring in the hospital and insisted on getting discharged against medical advice; however, she returned to the hospital within 24 hours with worsening distension and systemic inflammatory response signs. She was febrile and tachycardic with a pulse rate of 140/min. She remained stable haemodynamicaly and oxygenation-wise. A repeat CT imaging revealed pneumoperitoneum (Figures 4, 5].

**Figure 4:**
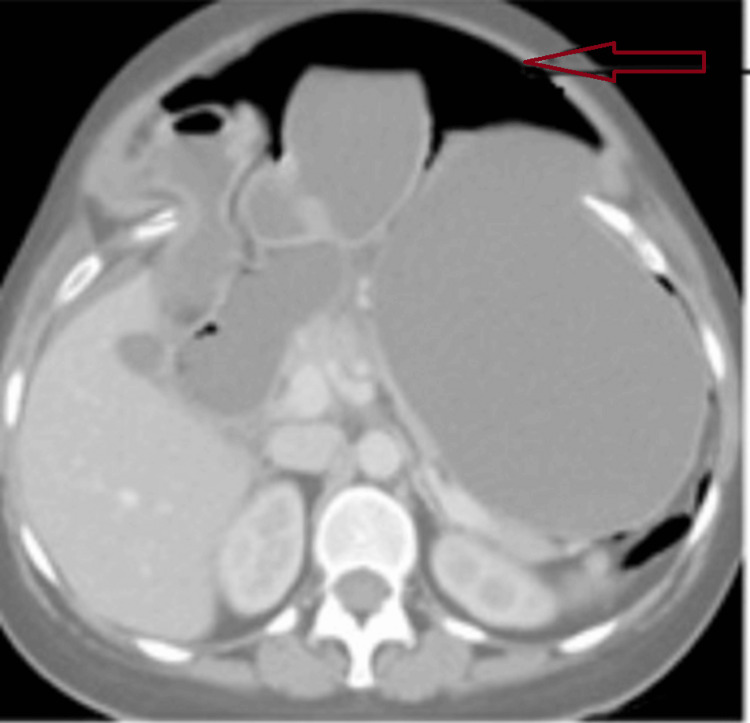
Repeat CT abdomen (axial view) on postpartum day 7 Dilated stomach and small bowel loops with pneumoperitoneum (red arrow).

**Figure 5:**
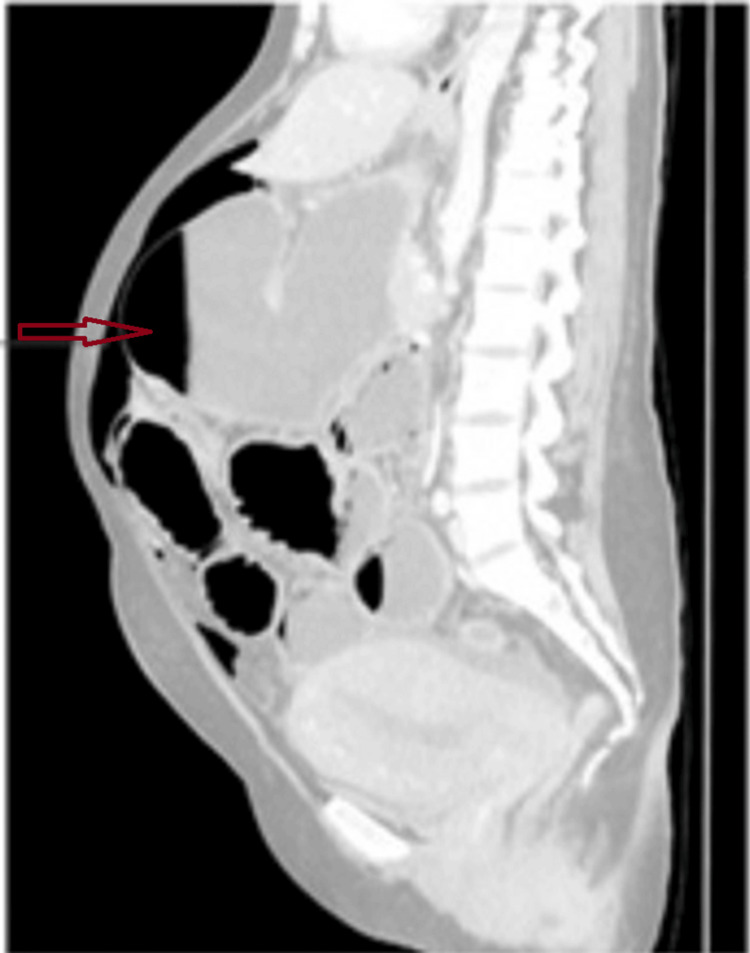
CT abdomen (sagittal view) The image demonstrates the pneumoperitoneum (red arrow) with a dilated stomach and small bowel loops.

Management and outcome

Emergency laparotomy revealed thin, flimsy adhesions (Figure 6) and a large cecal perforation (Figure 7). The patient underwent bowel resection and primary anastomosis with a diverting ileostomy.

**Figure 6:**
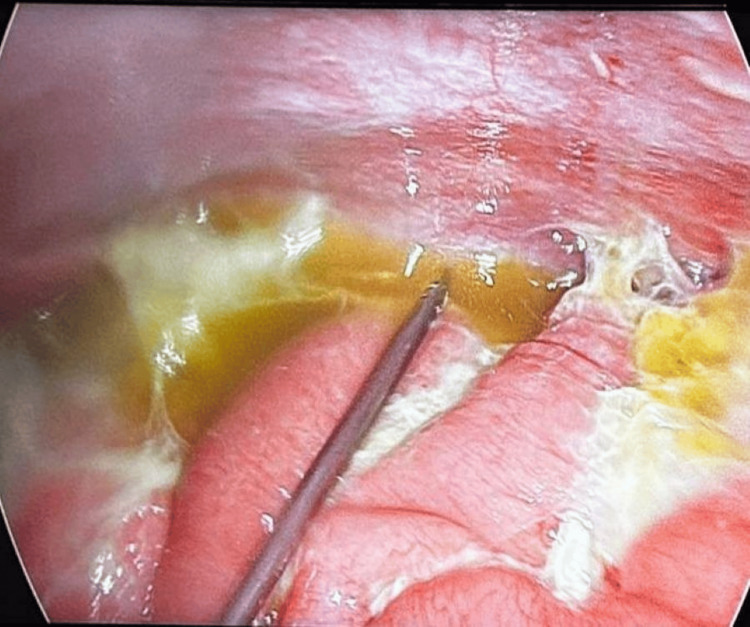
Intraoperative images showing thin adhesions

**Figure 7:**
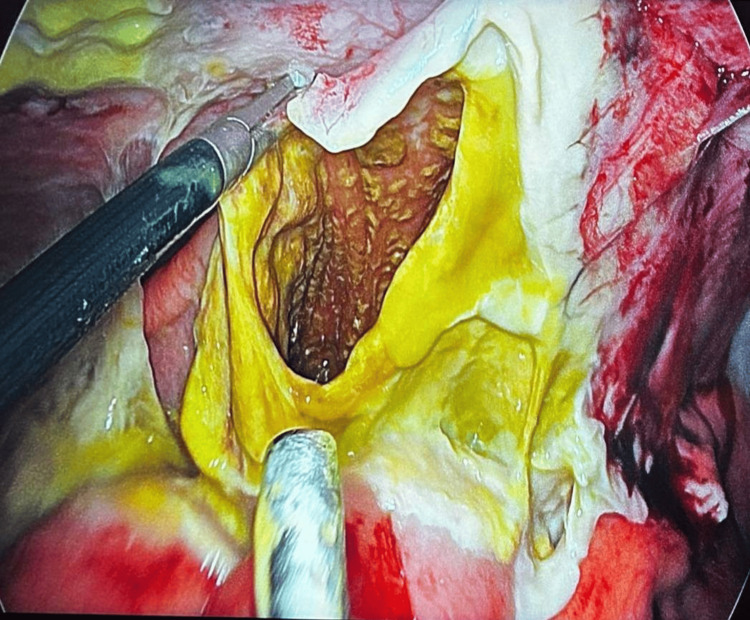
Intraoperative finding showing perforation of caecum and fecal contamination

Postoperatively, she remained hemodynamically stable with persisting fever spikes. The ileostomy was functional. Antibiotics were escalated to carbapenems, with Gram-positive coverage. Tissue biopsy grew carbapenem-resistant Enterobacteriaceae (CRE)- Escherichia coli, likely due to fecal contamination. Antibiotics were optimized. The clinical condition got better. Electrolytes were corrected. She was shifted back to the ward on postpartum day 3 and subsequently got discharged home with a functional ileostomy in a stable condition. Table 1 presents a summary of the patient's clinical timeline.

**Table 1:** Key events postpartum PPD: Postpartum day, SIRS: Systemic inflammatory response syndrome, NPO: *nil per os*, CRE: Carbapenem-resistant Enterobacteriaceae.

Postpartum Day	Clinical event	Key Findings
PPD 0	Delivery	Forceps-assisted vaginal delivery + Episiotomy.
PPD 1	Symptom onset	Severe bloating, abdominal pain.
PPD 2	"Improvement"	Bowels opened with medical management, NPO as per surgical advice
PPD 3	SIRS response	Fever spikes; abdomen distension persisted. CT imaging with oral contrast shows features of pseudo-colonic obstruction
PPD 9	Readmission	Fever spikes, tachycardia. Repeat CT abdomen with rectal contrast – Pneumoperitoneum Emergency Explorative Laparotomy- Cecal perforation; Resection + Ileostomy.
Post-bowel resection	Microbiology	CRE Escherichia coli isolated from tissue biopsy.

## Discussion

Ogilvie’s syndrome is an acute condition that is a temporary, non-mechanical obstruction mainly affecting the large bowel. The condition is named after Sir William Heneage Ogilvie [12]. It is most commonly encountered in elderly patients with comorbidities and a history of constipation. It is very common after abdominal surgeries, trauma, infection, neurological diseases, and immobilization. Postoperative Ogilvie syndrome develops over three to five days. Electrolyte imbalances are one of the major triggers. Jetmore et al., among their 48 cases of Ogilvie syndrome, noted that 83% demonstrated at least one electrolyte disturbance, with hypocalcemia being the most common [13].

Pregnancy, especially post-cesarean section, is one of the important and rare causes of Ogilvie syndrome. To the best of our knowledge, only three cases of pseudo-colonic obstruction with cecal perforation after normal vaginal delivery have been reported in the medical literature to date [14-16]. Possible risk factors in the postpartum period include prolonged labor, maternal fatigue, regional anesthesia, and operative deliveries [10,11]. However, the exact pathogenesis is unclear. Few studies attributed that to the reduced estrogen levels [17]. The mechanism could involve S2 to S4 parasympathetic nerves. Hormonal changes cause loss of tone of these nerves, which results in an atonic distal colon and pseudo- obstruction [18]. The area of dilated and collapsed bowel around the splenic flexure corresponds to the transition zone between the vagal and sacral parasympathetic nerve supply. A cut-off sign between these areas in the imaging is used to support the hypothesis of parasympathetic inhibition causing Ogilvie’s syndrome.

Diagnosis is often delayed due to nonspecific symptoms. The first and classical symptom is abdominal distension without passage of flatus. In a study done by Jetmore et al., no known case of Ogilvie syndrome presented without this symptom [13]. In our case, the patient was asymptomatic at the time of initial presentation for induction of labor, except for clear appreciation of fetal movements. The patient developed nausea, vomiting, and abdominal pain and bloating 12 hours after vaginal delivery. The cecal diameter in the initial CECT abdomen was 7.2 cm, though there was predominant dilation of the large bowel. She passed stools after 24 hours, likely due to the SOS prokinetics she had received in the meantime. Patients with bowel perforation can have fever, tachycardia, hypotension, and abdominal pain, all indicating intra-abdominal sepsis.

One of the differential diagnoses is paralytic ileus. Both have similar presentations and clinical implications. Gut motility impairment in paralytic ileus often has a rapid onset, developing within a day or two. It may progress to complete cessation of gut motor activity. Involvement of the entire gastrointestinal tract, including the esophagus, is noted in paralytic ileus, whereas Ogilvie’s syndrome is specific to the large intestine. Patients with Ogilvie’s syndrome take a longer time to recover than those with paralytic ileus.

Cecal anatomy plays an important role in its predisposition to perforation in Ogilvie’s syndrome. The cecum has the thinnest wall and largest diameter in the entire large intestine. It also has a competent ileocecal valve, which prevents the decompression of the cecum and proximal colon. The cecum expands three times faster than the rest of the colon. According to the law of Laplace, the tension in the wall of a hollow viscus is directly proportional to its radius and intraluminal pressure. Massive dilation of the colon leads to increased wall tension, causing the cecum to be the common site for ischemia and necrosis [19]. An erect abdominal X-ray is the first-line investigation and shows dilation of the bowel loops. Air under the diaphragm is often seen with a perforation. The investigation of choice is CT abdomen, possibly contrast-enhanced, which rules out mechanical obstruction and provides a better picture of cecal dilation and the nature of the bowel.

Cecal diameter is considered important in prognosis and management. Management includes medical, minimally invasive, and surgical options. Medical management-wise, the patient is kept nil per oral, and nasogastric decompression is done to empty the upper gastrointestinal contents. Ambulation, especially in postoperative cases, helps reinitiate normal gastrointestinal tract motility [5]. When the colonic dilation exceeds 9-10 cm, pharmacological therapy with neostigmine and colonic decompression with a large flatus tube may be useful [12]. The reported success rate for this technique ranges from 77% to 96% [20]. Minimally invasive options like colonoscopy decompression help in relieving the obstruction and restoring bowel motility. However, it has been reported that this approach carries a 15% failure risk [21]. Cecal diameter of 9 cm and above indicates imminent perforation [22]. Diagnostic laparotomy may help to prevent intra-abdominal sepsis in cases with signs of imminent perforation. Surgery is also required in extreme cases with signs of perforation and peritonitis.

## Conclusions

Cecal perforation secondary to post-partum Ogilvie syndrome remains a diagnostic pitfall, particularly following spontaneous or operative vaginal deliveries, where clinical suspicion remains generally lower compared to post-cesarean cases, given the absence of intra-abdominal instrumentation. In this case, the symptoms (abdominal distension and discomfort) are often mistaken for "post-birth discomfort, masking the perforation. While cesarean cases are the most common causes, vaginal delivery cases are the ones most likely to be missed. The return of flatus or bowel movements does not rule out the impending cecal perforation. Clinicians always have to communicate the risk of silent perforation to patients who feel "well enough" to go home despite unresolved physical findings. When cecal diameter approaches critical thresholds (>9 cm) or when systemic signs like fever spikes persist despite conservative measures, prompt surgical or endoscopic decompression is the only way to prevent high morbidity and mortality. This case serves as a stark reminder of several critical clinical "pearls". The take-home message here is that early diagnosis and timely intervention are essential to reduce mortality and morbidity.
